# Development of toileting behaviour evaluation for Japanese older patients using wheelchairs in a hospital setting: a validation study

**DOI:** 10.1186/s12877-023-04069-9

**Published:** 2023-06-06

**Authors:** Yasuhiro Higashi, Toshikatsu Kaneda, Yoshimi Yuri, Takumi Horimoto, Yuta Somei, Kimiaki Hirayama

**Affiliations:** 1 Faculty of Health Sciences, Morinomiya University of Medical Sciences, 1-26-16, Nankokita, Suminoe, Osaka, 559-8611 Japan; 2 Department of Rehabilitation, Kansai Rehabilitation Hospital, 3-11-1, Sakuranocho, 560-0054 Toyonaka, Osaka, Japan; 3 Department of Rehabilitation, Osaka General Hospital of West Japan Railway Company, 1-2-22, Matsuzakicho, 545-0053 Abeno, Osaka, Japan; 4 Department of Rehabilitation, Kiba Hospital, 4-2-8, Iwatacho, 578-0941 Higashiosaka, Osaka, Japan

**Keywords:** Toileting behaviour evaluation, Reliability, Validity, Activities of Daily Living

## Abstract

**Background:**

Ageing limits the activities of daily living (ADLs). Among ADLs, a lack of toileting independence causes a decline in the quality of life, mental health, and social participation. Therefore, occupational therapists spend considerable time assessing toileting disability based on various assessment methods for toileting behaviour. However, these assessment methods have issues with the grading levels, number of items, and diseases covered, and they fail to evaluate toileting behaviour accurately and sensitively. Hence, this study developed a Toileting Behaviour Evaluation (TBE) on a 6-point ordinal scale for patients using wheelchairs, with 22 activity components for various diseases.

**Methods:**

This study examined the reliability and validity of the TBE in acute and subacute hospitals in Japan. To this end, two occupational therapists assessed 50 patients for inter-rater reliability at different times and one assessed them twice within 7–10 days for intra-rater reliability using the TBE. Furthermore, occupational therapists assessed 100 patients for internal consistency using the TBE and for concurrent validity using the TBE and Functional Independence Measure (FIM). The patients had been diagnosed with various diseases. This study used the weighted kappa coefficient for statistical analysis of the inter-rater and intra-rater reliability, Cronbach’s alpha coefficient for internal consistency, and Spearman’s rank correlation coefficient for concurrent validity. We performed all statistical analyses using the IBM SPSS Statistics ver. 25 for Windows. All P-values < 0.05 were considered statistically significant.

**Results:**

The minimum weighted kappa coefficients for the inter-rater and intra-rater reliability for each item were 0.67 and 0.79, respectively. Cronbach’s alpha was 0.98 for the 22 items. The Spearman’s rank correlation coefficient between the mean scores on the TBE and FIM for toilet-related items was 0.74 (P < .01).

**Conclusions:**

The TBE demonstrated good reliability and validity. This means that therapists can use it to identify impaired toileting behaviour. However, the relationship between impairments and each item of toileting behaviour should be explored in future studies. Additionally, studies should examine the creation of a specific index of functions of independence in each toileting behaviour.

## Background

Globally, the number and proportion of people aged 60 years and older have increased rapidly. Indeed, this number is expected to increase from 1 billion in 2019 to 1.4 billion and 2.1 billion by 2030 and 2050, respectively [1].

In 2020, 28.6 per cent of the population in Japan was aged 65 years old and over, indicating that the ageing society in Japan is progressing quite rapidly compared to the US and the European countries [2].

Ageing limits the activities of daily living (ADLs) [3]. Specifically, it decreases physical function and increases morbidity [4], thereby impairing ADLs [5]. In Japan, health insurance plans that cover acute and recovery phase rehabilitation services were started in 2000. These services provide intensive rehabilitation to patients to help them regain the ability to perform ADLs [6, 7].

Among ADLs, toileting independence in particular impacts the length of stay in hospitals and the time of discharge [8]. For instance, previous studies have documented that independent toilet activity in hospitalized patients was highlighted as a key factor in determining the time of discharge [9–11]. A lack of toileting independence, notably, causes a decline in the quality of life [12], mental health, and social participation [13]. Studies have also shown that toileting dependence results in economic, mental, and physical burdens for not only the patients but also their families and society [14, 15]. Therefore, occupational therapists providing ADL support must assess and assist patients with toileting activities in acute and recovery phase rehabilitation services (subacute hospitals). The Functional Independence Measure (FIM) and the Barthel Index (BI) are international assessment methods that are widely used by occupational therapists to evaluate toileting behaviour. However, these instruments do not provide a breakdown of the individual component activities that comprise toileting behaviour and indicate poor responsiveness [16]. These component activities include putting on and taking off pants, wiping the buttocks, and tearing off toilet paper [17, 18]. An assessment of the individual components of each daily activity has been shown to be effective for determining rehabilitation goals and treatment planning [17]. Therefore, an evaluation of the component activities of toileting behaviour is necessary.

The following three methods are used to evaluate these component activities: the Toileting Performance Assessment Test (TPAT) [16], Toileting Tasks Assessment Form (TTAF) [17], and Toileting Assessment Test (TAT) [18]. The TPAT classifies the toileting activities of patients using wheelchairs into 10 component activities, such as transferring from bed to wheelchair, standing up, and shifting weight to one side while maintaining a standing position. It has three grading levels— (0) not possible, (1) possible with partial assistance, and (2) possible, and its reliability, validity, and responsiveness have been verified [16]. The TTAF classifies the toileting behaviour of patients using wheelchairs into 24 component activities, including opening and closing the toilet door, standing up from the wheelchair, and pulling the lower garments down. It has three grading levels—(A) independent, (B) requires supervision or verbal assistance, and (C) requires assistance, and research has verified its reliability and validity [17]. The TAT classifies the toileting behaviour of patients using wheelchairs into 10 component activities, such as standing up from the toilet, taking off pants, and wiping the buttocks. It has five grading levels: (0) no instruction, (1) verbal instruction, (2) modelling, (3) physical cueing, and (4) physical guidance. The validity of the TAT has been partially examined [18].

Although these evaluation methods can be used to achieve a sensitive and accurate evaluation of patients’ toileting behaviour, the limited number of grading levels in the TPAT and TTAF continue to pose a challenge for capturing small changes in patients. This highlights the need to incorporate more detailed grading levels to achieve a more precise evaluation. The TPAT and the TAT are also limited in that they classify toileting behaviour into only 10 component activities while omitting several major activities (“Open the door”, “Manoeuvre the wheelchair to the appropriate place for transfer to the toilet seat”,” Lock the wheelchair brakes”, “Place feet on the footrest”), so they do not cover the important components of toileting behaviour. Another common limitation of these three assessment methods is that they are designed to assess only stroke patients. These limitations highlight the need for an evaluation method that includes detailed grading levels, a larger number of component activities of toileting behaviour, and a wide range of diseases. Therefore, we developed the Toileting Behaviour Evaluation (TBE) using a 6-point ordinal scale comprising 22 component activities for patients using wheelchairs. This study examined the reliability and validity of the TBE for various diseases frequently managed by therapists.

## Methods

### Study sample and setting

To study the inter-rater and intra-rater reliability (test-retest reliability), we conducted the study at two hospitals in an urban area of Japan where we belong; these included acute and subacute hospitals that have an average number of beds and are staffed by rehabilitation specialists and occupational therapists. We enrolled participants who were admitted to these two hospitals between April 2020 and March 2021.

For internal consistency and concurrent validity as well, we conducted the study at three acute and subacute hospitals in an urban area of Japan where we belong. The selection criteria were the same as those of the reliability study. We enrolled participants who were admitted to these three hospitals between April 2020 and March 2021.

Participants were selected if they were aged 60 years or above and able to sit in a wheelchair. We excluded participants if they were physically unable to perform toileting activities (e.g. people with bladder catheters), could walk to the toilet, or could not give consent. For inter- and intra-rater reliability, we also excluded those whose FIM toilet-related behaviour scores changed during the study period. Within each hospital, therapists randomly selected subjects who met the above criteria. Following the COnsensus-based Standards for the selection of health status Measurement INstruments (COSMIN) Study Design checklist, we aimed to have a sample size of 50 for inter-rater and intra-rater reliability and 100 for internal consistency and concurrent validity [19]. The COSMIN Study Design checklist is recommended for use by researchers and clinicians or other professionals designing studies to evaluate the measurement properties of existing patient-reported outcome measures (PROMs). In this checklist, each standard is accompanied by a 4-point rating scale. This 4-point rating scale is added for illustrative purposes to better understand the consequences of choices made in a study design for the methodological quality of the study.

### Assessment

#### Toileting behaviour evaluation (TBE)

Between April 2019 and January 2020, we conducted five meetings with six occupational therapists holding a master’s degree or higher, where we reviewed items from preceding studies and analysed the toileting behaviour of patients using wheelchairs. Initially, a scale consisting of 22 items on a 4-point scale (4: independence, 3: supervision, 2: verbal assistance, 1: physical assistance) was developed. It was then reviewed for clinical utility, and at the meeting with the six experts, the scale was revised to 22 items on a 6-point scale (6: independence, 5: modified independence, 4: supervision, 3: verbal assistance, 2: physical assistance, and 1: total assistance). This scale was again used in clinical practice with several patients to confirm its practicality, and all six experts agreed on this scale at a meeting. Finally, we developed the TBE with 22 items on a 6-point ordinal scale (Appendix 1). The evaluation procedure involved observing the sequence of the participants’ toileting behaviour. Following an earlier study [18], we scored the participants by the level of assistance they required for toileting to evaluate their maximum ability. In other words, we provided the scoring in the following order: ‘independence’, ‘modified independence’, ‘supervision’, ‘verbal assistance’, ‘physical assistance’, and ‘total assistance’. Physical assistance does not precede verbal assistance, except in emergency situations. Verbal assistance includes gestures (e.g. pointing gestures) and is different from supervision.

**Appendix 1:** Toileting Behaviour Evaluation (TBE)

Item	Score	Comments	Factor
Open the door			
Close the door			
Turn on the light			
Manoeuvre the wheelchair to the appropriate place for transfer to the toilet seat			
Lock the wheelchair brakes			
Take the footrests up			
Stand up from the wheelchair			
Turn while standing			
Maintain a standing position			
Pull the lower garments down			
Sit on the toilet seat			
Maintain a sitting position on the toilet seat			
Clean up after urination and/or defecation with toilet paper			
Stand up from the toilet seat			
Maintain a standing position			
Pull the lower garments up			
Turn while standing			
Sit on the wheelchair seat			
Place feet on the footrest			
Unlock the wheelchair brakes			
Flush the toilet			
Open the door and exit the toilet room			

Note. 6: Independence 5: Modified Independence 4: Supervision 3: Verbal Assistance 2: Physical Assistance 1: Total Assistance

#### Functional independence measure (FIM)

Developed by Granger in 1983, the FIM is an ADL assessment method measuring a person’s level of disability in terms of the burden of care. It covers two broad functional domains: motor and cognition. The FIM-Motor comprises 13 items based on 4 domains (self-care, sphincter control, transfers, locomotion). The FIM-Cognition comprises 5 items based on 2 domains (communication and social cognition). Each FIM item is scored on a 7-point ordinal scale ranging from total assistance (or complete dependence) to complete independence. There are two toilet-related items—toilet transfer (involving movement between the wheelchair and the toilet seat) and toileting (involving maintaining perineal hygiene and adjusting clothing before and after using a toilet).

### Reliability

We adopted the following procedure to conduct the inter-rater reliability assessment. First, we randomly chose two occupational therapists on each occasion to assess the participants at different times using the TBE (the second occupational therapist assessed the participant within 10 days of the assessment by the first occupational therapist). Second, we examined the rate of agreement between the results of each assessment.

For intra-rater reliability, we chose one occupational therapist at random to assess the participants using the TBE twice within a period of 7 to 10 days. Subsequently, we examined the rate of agreement between the results of each assessment. To analyse the assessments, we calculated the weighted kappa coefficients for each of the 22 items for both inter-rater and intra-rater reliability.

### Internal consistency

The participants were evaluated using the TBE, and the Cronbach’s alpha was calculated based on the item scores of all the participants.

### Concurrent validity

We conducted two assessments to examine concurrent validity. Occupational therapists assessed the participants using the TBE and the FIM within 10 days of each other. For the analysis, we calculated the correlation coefficients between the mean scores of the TBE and the FIM toilet-related item scores (e.g. toilet movement and toilet transfer). We also calculated the correlation coefficients between the scores of each item of the TBE and the FIM toilet-related item scores.

### Participants’ information

We collected participants’ demographic characteristics (e.g. age, gender), diagnosis, and FIM scores from their medical records.

### Statistical analysis

We used the weighted kappa coefficient for each of the 22 items for statistical analysis of the inter-rater and intra-rater reliability, Cronbach’s alpha coefficient for internal consistency based on the item scores of all the participants, and Spearman’s rank correlation coefficient for concurrent validity. We performed all statistical analyses using the IBM SPSS Statistics ver. 25 for Windows. All P-values < 0.05 were considered statistically significant.

## Results

### Reliability

For inter-rater reliability, we sampled 50 (18 men, 32 women) patients with a mean age of 79.1 years (SD = 8.4). Table 1 shows the participant characteristics, including their FIM-Motor and FIM-Cognition information (Table 2). A total of 34 occupational therapists (range of experience: 1–17 years; mean = 5.7 years, SD = 4.6) participated in this study, and the mean difference in years of experience between two paired occupational therapists was 5.4 years (SD = 3.9). The mean weighted kappa coefficient for each item was 0.74 (range: 0.68–0.85; Table 1), and all the coefficients were significant.

**Table 1:** Weighted kappa coefficient of each item of the Toileting Behaviour Evaluation

Item	Inter-rater weighted κ	Intra-rater weighted κ
Open the door	0.75	0.87
Close the door	0.75	0.95
Turn on the light	0.72	0.86
Manoeuvre the wheelchair to the appropriate place for transfer to the toilet seat	0.70	0.89
Lock the wheelchair brakes	0.78	0.93
Take the footrests up	0.73	0.96
Stand up from the wheelchair	0.75	0.92
Turn while standing	0.76	0.94
Maintain a standing position	0.70	0.81
Pull the lower garments down	0.77	0.85
Sit on the toilet seat	0.71	0.91
Maintain a sitting position on the toilet seat	0.75	0.93
Clean up after urination and/or defecation with toilet paper	0.69	0.91
Stand up from the toilet seat	0.69	0.91
Maintain a standing position	0.68	0.82
Pull the lower garments up	0.79	0.78
Turn while standing	0.77	0.92
Sit on the wheelchair seat	0.77	0.92
Place feet on the footrest	0.79	0.95
Unlock the wheelchair brakes	0.79	0.95
Flush the toilet	0.85	0.97
Open the door and exit the toilet room	0.69	0.85
AVERAGE	0.74	0.90

For intra-rater reliability, we sampled 50 participants (20 men, 30 women) with a mean age of 78.2 years (SD = 8.4) (Table 2). A total of 22 occupational therapists (range of experience: 1–17 years; mean = 6.0 years, SD = 4.7) participated in this study. The mean weighted kappa coefficient for each item was 0.90 (range: 0.78–0.97) (Table 1), and all the coefficients were significant.

**Table 2:** Participants’ characteristics

		Inter-rater Reliability	Intra-rater Reliability	Internal Consistency/ Concurrent Validity
Number		50	50	100
Age				
	Years, mean (SD)	79.1 (8.4)	78.2 (8.4)	79.0 (8.0)
	Range	62–100	60–100	60–100
Gender				
	Male	18	20	50
	Female	32	30	50
Diagnosis				
	Cerebrovascular Accident	38	36	71
	Circulatory System	0	0	1
	Musculoskeletal System and Connective Tissue	8	9	21
	Nervous System	2	1	4
	Respiratory System	0	0	1
	Kidney and Urinary Tract	2	2	2
	Injuries, Poison, and Toxic Effects of Drugs	1	2	3
FIM-Motor				
	Mean (SD)	42.0 (14.6)	44.6 (22.1)	45.5 (15.8)
	Range	15–73	15–75	15–77
FIM-Cognition				
	Mean (SD)	21.4 (7.7)	15.4 (7.9)	21.3 (7.4)
	Range	8–35	8–35	8–35

### Internal consistency

We sampled 100 participants (50 men, 50 women) with a mean age of 79.0 years (SD = 8.00). Table 1 shows the participant characteristics, including their FIM-Motor and FIM-Cognition information (Table 2). A total of 39 occupational therapists (range of experience: 1–19 years; mean = 6.2 years, SD = 4.9) participated in this study. Cronbach’s alpha was 0.98 for the 22 items.

### Concurrent validity

The participants and the occupational therapists were the same as that for the internal consistency study (Table 2). The Spearman’s correlation coefficient between the mean scores on the TBE and the FIM toilet-related item scores was 0.74 (P < .01). All the correlations showed significance. The correlation coefficients between each item score and the FIM toilet-related item scores were 0.57–0.79 (Table 3). All correlations were significant.

**Table 3:** Correlation between each item of the Toileting Behaviour Evaluation and Functional Independence Measure ‘toileting’ scores

	FIM ‘toileting’ scores
Open the door	0.59
Close the door	0.61
Turn on the light	0.62
Manoeuvre the wheelchair to the appropriate place for transfer to the toilet seat	0.63
Lock the wheelchair brakes	0.63
Take the footrests up	0.70
Stand up from the wheelchair	0.71
Turn while standing	0.76
Maintain a standing position	0.68
Pull the lower garments down	0.79
Sit on the toilet seat	0.73
Maintain a sitting position on the toilet seat	0.57
Clean up after urination and/or defecation with toilet paper	0.66
Stand up from the toilet seat	0.64
Maintain a standing position	0.68
Pull the lower garments up	0.79
Turn while standing	0.75
Sit on the wheelchair seat	0.76
Place feet on the footrest	0.68
Unlock the wheelchair brakes	0.64
Flush the toilet	0.67
Open the door and exit the toilet room	0.57

## Discussion

In this study, the TBE was developed to assess the toileting ability of patients with various diseases. The target sample size was reached for all analyses, and the COSMIN checklist criteria [19] were met adequately or higher. We believe that the method and procedures were followed rigorously and were appropriate.

For reliability research, kappa coefficients were interpreted as follows: ≤ 0.20, 0.21–0.40, 0.41–0.60, 0.61–0.80, and ≥0.81 as slight, fair, moderate, substantial, and almost perfect agreement, respectively [20]. The minimum kappa coefficient for inter-rater reliability in this study was 0.68, indicating at least substantial inter-rater reliability for each TBE item. The inter-rater reliability of the mean score was also substantial at 0.74. This value suggests that similar results were obtained when two occupational therapists with different years of experience evaluated the same patients. The intra-rater reliability of the mean score was almost perfect at 0.90, and the minimum kappa coefficient was 0.78, indicating at least substantial intra-rater reliability for each TBE item. It means the TBE was a reproducible assessment. Cronbach’s alpha was 0.98, indicating almost perfect internal consistency. The TBE could measure the ability to perform toilet activities as a whole. The correlation coefficients between the mean score of the TBE and the FIM toilet-related item scores were more than 0.70, indicating a high correlation. It indicated that the TBE was a valid assessment method for toileting abilities; it had good inter- and intra-rater reliability, internal consistency, and concurrent validity.

Previous studies reported the minimum weighted kappa coefficient value for inter-rater reliability of the TPAT to be the same as that of the TTAF (0.61) [16, 17]. For intra-rater reliability, minimum weighted kappa coefficient values were reported at 0.83 [16] and 0.60 [17] for the TPAT and the TTAF, respectively, similar to those in this study. They also reported Cronbach’s alpha values of 0.97 for the TPAT [16] and 0.95 or 0.94 for the TTAF [17], which are also similar to the current results. The previous concurrent validity study reported a correlation coefficient value of 0.86 between the TPAT and the FIM total score [16] and correlation coefficients of 0.88–0.93 and 0.91–0.93 between the TTAF and the FIM toileting scores and toilet transfer scores, respectively [17]. These results are also similar to the current findings. Additionally, this study found a moderate and higher correlation between the scores of each TBE item and the FIM toilet-related item scores. We found that the correlation coefficients were below 0.60 for ‘open the door’, ‘close the door’, ‘turn on the light’, ‘manoeuvre the wheelchair to the appropriate place for transfer to the toilet seat’, ‘maintain a seating position on the toilet seat’, and ‘open the door and exit the toilet room’. These items might show low correlations because they were not assessed under the transfer and toileting items of the FIM. However, the items covered by the FIM showed correlations of 0.70 or higher. In other words, the validity of each item of the TBE was confirmed.

With 22 items on a 6-point ordinal scale, the TBE could be used to evaluate in detail the toileting behaviour of patients with various diseases, who are frequently evaluated by therapists in the hospital for toileting. Despite an increase in the total number of items as well as the values on the rating scale, inter- and intra-rater reliabilities were high. The inter-rater reliability study yielded particularly interesting results. The mean difference in the raters’ years of therapist experience was 5.4 years, showing that, despite the difference in the years of experience, therapists can provide similar evaluations of the participants’ toileting behaviour using the TBE. As mentioned in the method to assess participants’ maximum ability, the assistance provided was scored in the following order: independence, modified independence, supervision, verbal assistance, physical assistance, and total assistance. Physical assistance did not precede verbal assistance. The verbal assistance included gestural prompting (e.g. pointing gesture), which was distinguished from supervision. Since these criteria were clear and simple, it was easy for the therapists to conduct the evaluation.

### Limitations and future studies

The use of the TBE could help therapists identify which part of the toileting sequence poses a challenge to the participants. However, the study’s target population comprised those admitted to acute or subacute hospitals. Therefore, further research is needed before it can be used for patients with other diseases admitted to nursing homes and other facilities. Investigating the causes hindering the independence of each item of toileting behaviour would be critical to provide more effective support. Hence, in future studies, we plan to examine the relationship between impairments and each item of toileting behaviour using the TBE.

## Conclusion

This study confirmed the reliability and validity of the TBE, designed to help identify impaired toileting behaviour. Future studies should explore the relationship between functional disability and each item of toileting behaviour and discuss the creation of a specific index of functions for achieving independence in each toileting behaviour.

## Data Availability

The datasets generated and/or analysed during the current study are not publicly available due to ethical reasons but are available from the corresponding author upon reasonable request.

## List of abbreviations

ADL: activities of daily living
BI: Barthel Index
FIM: Functional Independence Measure
TPAT: Toileting Performance Assessment Test
TTAF: Toileting Tasks Assessment Form
TAT: Toileting Assessment Test
TBE: Toileting Behaviour Evaluation
